# Taipei's Use of a Multi-Channel Mass Risk Communication Program to Rapidly Reverse an Epidemic of Highly Communicable Disease

**DOI:** 10.1371/journal.pone.0007962

**Published:** 2009-11-23

**Authors:** Muh-Yong Yen, Tsung-Shu Joseph Wu, Allen Wen-Hsiang Chiu, Wing-Wai Wong, Po-En Wang, Ta-Chien Chan, Chwan-Chuen King

**Affiliations:** 1 Department of Disease Control and Prevention, Taipei City Hospital, Taipei City Government, Taipei, Taiwan; 2 Institute of Emergency and Critical Care Medicine, National Yang-Ming University, Taipei, Taiwan; 3 Institute of Epidemiology, College of Public Health, National Taiwan University, Taipei, Taiwan; 4 Luke International Norway, Nøtterøy, Norway; 5 Department of Urology, National Yang-Ming University, Taipei, Taiwan; 6 Department of Health, Keelung City Government, Keelung, Taiwan; Columbia University, United States of America

## Abstract

**Background:**

In September 2007, an outbreak of acute hemorrhagic conjunctivitis (AHC) occurred in Keelung City and spread to Taipei City. In response to the epidemic, a new crisis management program was implemented and tested in Taipei.

**Methodology and Principal Findings:**

Having noticed that transmission surged on weekends during the Keelung epidemic, Taipei City launched a multi-channel mass risk communications program that included short message service (SMS) messages sent directly to approximately 2.2 million Taipei residents on Friday, October 12th, 2007. The public was told to keep symptomatic students from schools and was provided guidelines for preventing the spread of the disease at home. Epidemiological characteristics of Taipei's outbreak were analyzed from 461 sampled AHC cases. Median time from exposure to onset of the disease was 1 day. This was significantly shorter for cases occurring in family clusters than in class clusters (mean±SD: 2.6±3.2 *vs.* 4.39±4.82 days, p = 0.03), as well as for cases occurring in larger family clusters as opposed to smaller ones (1.2±1.7 days *vs.* 3.9±4.0 days, p<0.01). Taipei's program had a significant impact on patient compliance. Home confinement of symptomatic children increased from 10% to 60% (p<0.05) and helped curb the spread of AHC. Taipei experienced a rapid decrease in AHC cases between the Friday of the SMS announcement and the following Monday, October 15, (0.70% vs. 0.36%). By October 26, AHC cases reduced to 0.01%. The success of this risk communication program in Taipei (as compared to Keelung) is further reflected through rapid improvements in three epidemic indicators: (1) significantly lower crude attack rates (1.95% *vs.* 14.92%, p<0.001), (2) a short epidemic period of AHC (13 *vs.* 34 days), and (3) a quick drop in risk level (1∼2 weeks) in Taipei districts that border Keelung (the original domestic epicenter).

**Conclusions and Significance:**

The timely launch of this systematic, communication-based intervention proved effective at preventing a dangerous spike in AHC and was able to bring this high-risk disease under control. We recommend that public health officials incorporate similar methods into existing guidelines for preventing pandemic influenza and other emerging infectious diseases.

## Introduction

The viral illness known as acute hemorrhagic conjunctivitis (AHC) is frequently accompanied by a highly transmissible acute eye infection. This infection is most often caused by the adenovirus, enterovirus 70, and Coxsackie's virus. Coxsackie A24 infection was first reported in 1969 in Ghana and has since appeared around the world [1], [2], [3], [4], [5].

Since late 2002, several AHC epidemics have occurred in Asian countries such as Korea, Malaysia, and Singapore [6], [7], [8]. In September 2007, southern China's Coxsackie A24 AHC epidemic spread to Hong Kong after first appearing on the mainland in early summer. On September 18th, a cluster of cases with AHC-like symptoms was first unofficially reported by the media in Keelung, a harbor city bordering Taipei (816 km away from Hong Kong), in northern Taiwan. Because AHC was not on Taiwan's list of reported communicable diseases at the time, it was difficult for public health officials to collect adequate epidemiological data until October 4th, when a dramatic spike in AHC cases was reported by the mass media. At that time, the Keelung Department of Health reported 2722 cases of pink eye disease among public school students. Although general control measures were taken, case numbers continued to increase rapidly in Keelung, particularly during weekends.

**Figure 1:**
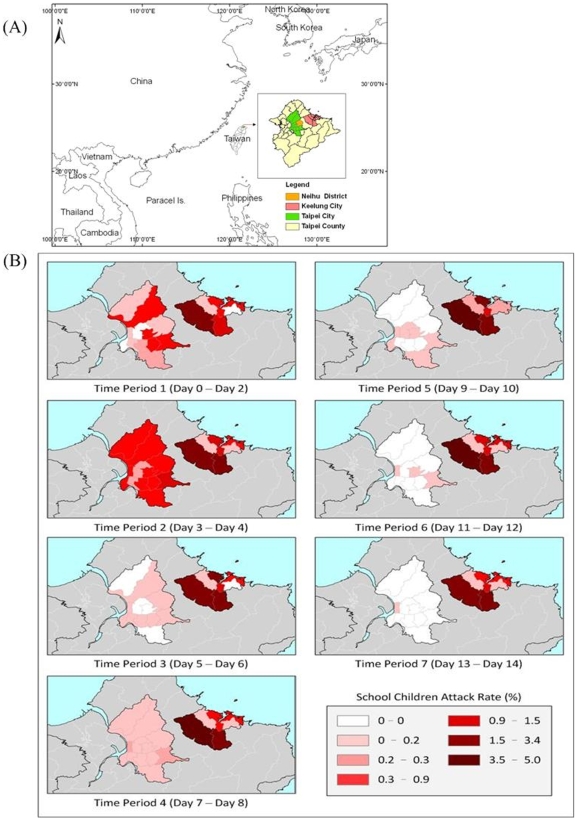
Spatial Distribution of the 2007 AHC Attack Rates in Taipei City and Keelung City. Geographical location of Taipei City and Keelung City and their spatial relationship was shown in panel A. Spatial and temporal changes of AHC attack rates (AR) between Taipei City and Keelung City were plotted according to their place in the outbreak timeline (panel B). Day 0 is used to indicate the days that cluster cases were first identified in Taipei or in Keelung cities. Darker colored areas indicate regions with higher AR.

Also on Oct. 4th, Taipei City Department of Health was alerted to its first AHC cluster (20 cases from a primary school) in Neihu, a Taipei district neighboring Keelung (Figure 1). Taipei was more prepared for the AHC outbreak as its alertness was raised by media coverage of outbreaks in nearby Keelung. The local Department of Health was also able to identify clusters quickly because it had made school reporting of influenza-like illness clusters (or other unusual clinical presentations) a mandatory practice since 2003.

Taipei's Department of Health had reorganized its disease control and prevention system against emerging infectious diseases (EID) after 2003's severe acute respiratory syndrome (SARS) epidemic but had not yet tested the system under real life circumstances. In launching countermeasures to bring the AHC outbreak under control, the Taipei Department of Health was also able to test its system and fine-tune its public health response for future EIDs. To evaluate the effectiveness of these intervention measures, daily surveillance was conducted to analyze the incidence rate and temporal-spatial distribution of new AHC cases. In addition, students' parents were sent questionnaires to capture their experience with these preventative measures.

## Materials and Methods

### Setting

Taipei City, the largest city in Taiwan, is located in northern Taiwan and directly borders Keelung City (Figure 1). In 2007, Taipei, a city of 2.6 million people occupying 271.8 km^2^, had a student population of 277,159 (10.53%). Taipei City has a larger population size and density than neighboring Keelung City (Table 1), and is, thus, exceedingly vulnerable to microbial transmission and EID outbreaks.

**Table 1:** General Information on the AHC Outbreak in Taipei City and Keelung City in 2007.

Demographic Information	Taipei City	Keelung City
**Duration of the AHC Outbreak (days)**	13	34
**Population Size**		
Reported AHC Cases in School Children	5,414	6,154
Total Number of School Children	277,159	41,244
Crude Attack Rate (%)*	1.95%	14.92%
Total Population	2,632,242	390,084
Population Density (per **Km^2^**)	9684.5	2937.4
**Geographic Area (Km^2^)**	271.8	132.8

*p **<0.001.**

### Ethics

This study was approved by the Advisory Committee for Infectious Diseases Control of Taipei City's Department of Health, Taipei City Government. Informed consent was obtained from the parents of participating schoolchildren in writing before they were asked to complete the questionnaire (File S1).

### Surveillance Methods

In response to a large outbreak of SARS in 2003, Taipei's Department of Heath established a new crisis management system with the goals of detecting EIDs early, implementing appropriate and timely public health responses, and administering effective risk management [9], [10]. At the start of Taiwan's 2007 AHC outbreak, local health departments in all of Taiwan's cities were engaged in a passive surveillance system in which schools reported cases as they occurred. On October 4th, Taipei's Department of Health urged schools and kindergartens with clusters of more than three AHC cases to actively report cases to the local Department of Health twice weekly. Age-specific incidence rates of AHC among school-aged children during the outbreak were also calculated using the collected surveillance data.

When total AHC cases in Taipei reached close to 500 on October 11, 2007, an active school-based surveillance system was launched by the city to monitor trends in the spread of AHC and evaluate the effectiveness of intervention measures. Schools were required to make daily reports to Taipei's Department of Education, regardless of whether there were any new cases on a given day. As the incidence rate of the last reported AHC cases in Keelung (on October 22) was 0.14%, we set this rate as a cut-off point for comparing the effectiveness of the countermeasures used by the two cities. Tailing data, *i.e.,* data that fell below the 0.14% incidence rate was excluded from our analysis. We used this cut-off to define the duration of the AHC epidemics for both cities. According to our definition, the AHC epidemics for each city began from the date of the first reported cluster and ended on the date that rates of new case incidence dropped to 0.14% or below.

### Epidemiological Design and Data Analysis

#### 1. Study subjects

We analyzed 461 reported AHC cases from ten Taipei schools (totaling 18,134 students). Students from six of the ten schools, three elementary schools and three junior high schools, made up most of the reported AHC case numbers. Two elementary schools and two junior high schools that were also included in the research reported continuous AHC occurrences during the week that our field epidemiological study was conducted. For each of the ten schools, we randomly selected one class that had had occurrences of AHC and one class where there had been no occurrence of AHC as our case and control groups, respectively. Data collected from these individual school groupings were gathered and divided into one study and one control group for data analysis.

#### 2. Case definition of AHC and clusters (family, school)

The AHC cases in this study all involved an acute conjunctiva inflammation that included eye redness (pink eye) accompanied with pain, swelling, tearing, or discharge from one or both eyes. Family clusters were defined as two or more cases of AHC occurring in one family within 14 days. Class clusters were defined as three or more AHC cases in one class of students within 7 days. Both family clusters and class clusters were charted through epidemiological investigation.

#### 3. Tempo-spatial data analysis

District-specific AHC attack rates were calculated by dividing the number of reported cases for each district by the total number of schoolchildren under its supervision. The Kriging method [11], [12], a statistical mapping technique utilizing data collected at each location, was used to interpolate each grid cell over a spatial domain. In this study, the centroid of each grid designated the attack rate in each district. In order to observe spatio-temporal spreading, Kriging assessed the spatio-temporal interactions in a diffusion map. We made a surface plot of daily time series as a gradient interpolated between adjacent days and district data points. Distance in the map symbolizes relative geographic relationship rather than actual distance. Townships were ordered from East to West and from North to South.

### Control Measures Used by Taipei City

To identify the possible etiologic agent of the outbreak, health care professionals were required to administer eye swabs and conduct laboratory testing. Local health workers were then able to match the epidemiological characteristics for the confirmed agent, Coxsackie A24, with appropriate and specific prevention and control measures. Government officials of the Taipei City Department of Health informed the media of the outbreak through press releases and used a variety of health education methods to reach the public. Beginning on October 5, public service messages were delivered to kindergartens, primary schools, middle schools, and high schools to encourage children to avoid touching their eyes, wash their hands routinely, and participate in disinfecting the school environment. Within schools, health education programming during daily morning assemblies provided updates to students and teachers regarding the current status of the epidemic and additional measures that were needed to reduce infections. A special telephone hotline was also established to improve case reporting and provide up-to-date disease counseling from health care institutions.

These measures, though helpful, had also been used in Keelung and had, thus far, proven inadequate at containing the epidemic. In early October 2007, Taipei City government decided to adopt a more aggressive campaign against the epidemic by implementing an “incident management system.” This system required various administrative agencies to follow an integrated disaster response plan. Although school closures and class cancellation were not required for schools with reported AHC cases, schools were encouraged to persuade symptomatic students to stay home and provide guidance on how to prevent the spread of the infection in the home environment. In addition, schools were authorized to keep symptomatic students from entering the school in case parents insisted on their attendance. If infected students were able to gain entry to school premises, the school was authorized to prevent them from joining public activities (such as swimming). School absenteeism was also reported and recorded daily.

The Taipei City Department of Health devised backup plans should the above-mentioned measures not succeed. These plans included separate care facilities for AHC patients at ophthalmic clinics, more intensive segregation of symptomatic students from classmates (in the classroom, at public washbasins, and during outdoor student activities), and quarantines that required symptomatic students to stay at home for seven days.

### Mass Risk Communication Program (MRCP)

**Figure 2:**
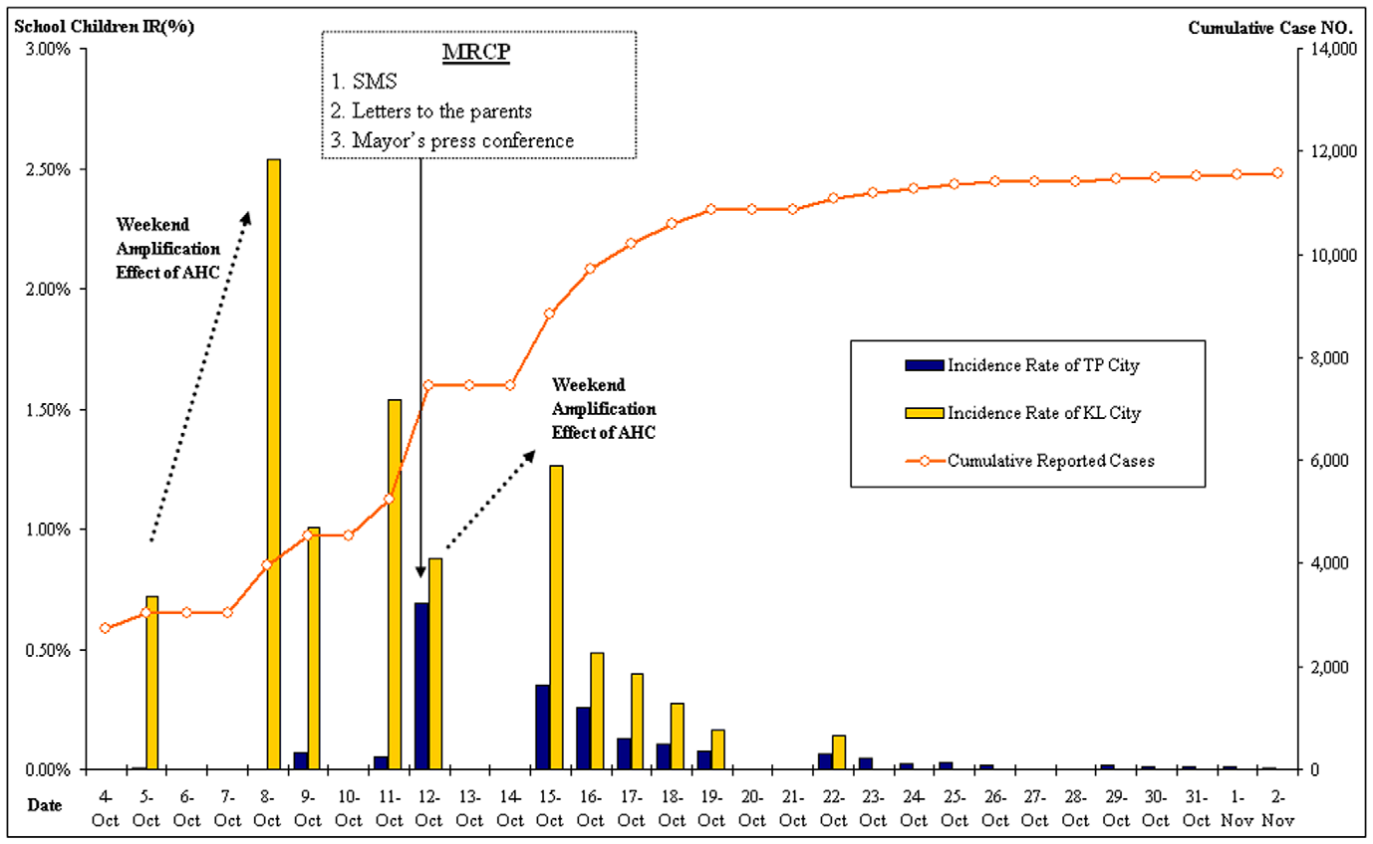
Incidence Rate (IR) and Cumulative Number of AHC Cases among School Children in Taipei City and Keelung City, October - November 2007. The figure's arrow indicates the weekend in which children stayed at home instead of attending school. The MRCP was launched on October 12th in Taipei City, causing the incidence rate (IR) of AHC to decline more rapidly in Taipei City than in Keelung City, where AHC cases continued to increase.

Monday incidence reports (Figure 2) exhibited tremendous increases in AHC case incidence during weekends in Keelung. Mindful of these weekend spikes, Taipei implemented a multi-channel risk communication prevention program during the weekend of Friday, October 12 (2,253 new cases of AHC were reported in Taipei on that date). This risk communication program focused on communicating directly to the public through three routes: (1) schools delivered a Taipei Department of Health letter signed by the mayor (that detailed AHC information and prevention methods) for students to take home to their parents, (2) the mayor held a press conference to discuss the epidemic and offer guidance to citizens for preventing the spread of the disease, and (3) over 2.2 million short message services (SMS) messages, a communication tool for exchanging short text messages between mobile telephonic devices, were delivered to all Taipei mobile phone numbers. The messages briefed Taipei residents on the current status of the epidemic and recommended citizen-level control measures. All communications suggested that symptomatic students stay at home, apart from other members of the family, and recommended household disinfection.

### Evaluating the Effectiveness of the Control Measures

On October 31st, all Taipei students involved in the study were asked to give their parents a questionnaire devised to collect epidemiological data and assess their opinions regarding Taipei City's infection control measures. The questionnaire asked about the clinical symptoms/signs of children with AHC, school attendance, and inquired on parents' sources of disease prevention information. Parents were also asked to comment on their degree of satisfaction with Taipei City's public health efforts and provide suggestions for improving future SMS alerts.

We evaluated the effectiveness of the control measures based on the duration of the epidemic and the attack rate of the disease among school students in both Taipei and Keelung. As Keelung was without a risk communications program, and had only applied the general control measures recommended by Taiwan's Center for Disease Control (Taiwan CDC), the effectiveness of the special measures taken by Taipei City could be readily calculated through direct comparison.

## Results

Because this study utilized risk communication methods used to minimize the public health threat of an unusual outbreak, we briefly describe the 2007 AHC epidemic below and analyze the effectiveness of the chosen methods using epidemiological measures.

### A. The 2007 AHC Epidemic

#### 1. AHC attack rates in Taipei and Keelung Cities

At the beginning of the epidemic, the etiologic agent of this AHC outbreak was unknown. On October 12th, based on culture and sequencing analysis performed at Taiwan CDC, the pathogen was identified as the A24 variant of the Coxsackie virus [13]. The epidemic lasted 13 days in Taipei (5,414 cases), and 34 days in Keelung (6,154 cases) (Table 1). Keelung City applied general control measures and Keelung CDC monitored daily reported new cases as suggested by Taiwan's CDC. However, there was no further assessment by Keelung on the effectiveness of its control measures. The crude attack rate of AHC in Keelung was significantly higher than in Taipei (14.92% *vs.* 1.95%; p<0.001). After the risk communication program was implemented in Taipei the overall incidence in the city decreased significantly (0.093% before *vs.* 0.056% after; p<0.001) while the incidence rates in Keelung continued to increase almost every weekend (Figure 2).

**Figure 3:**
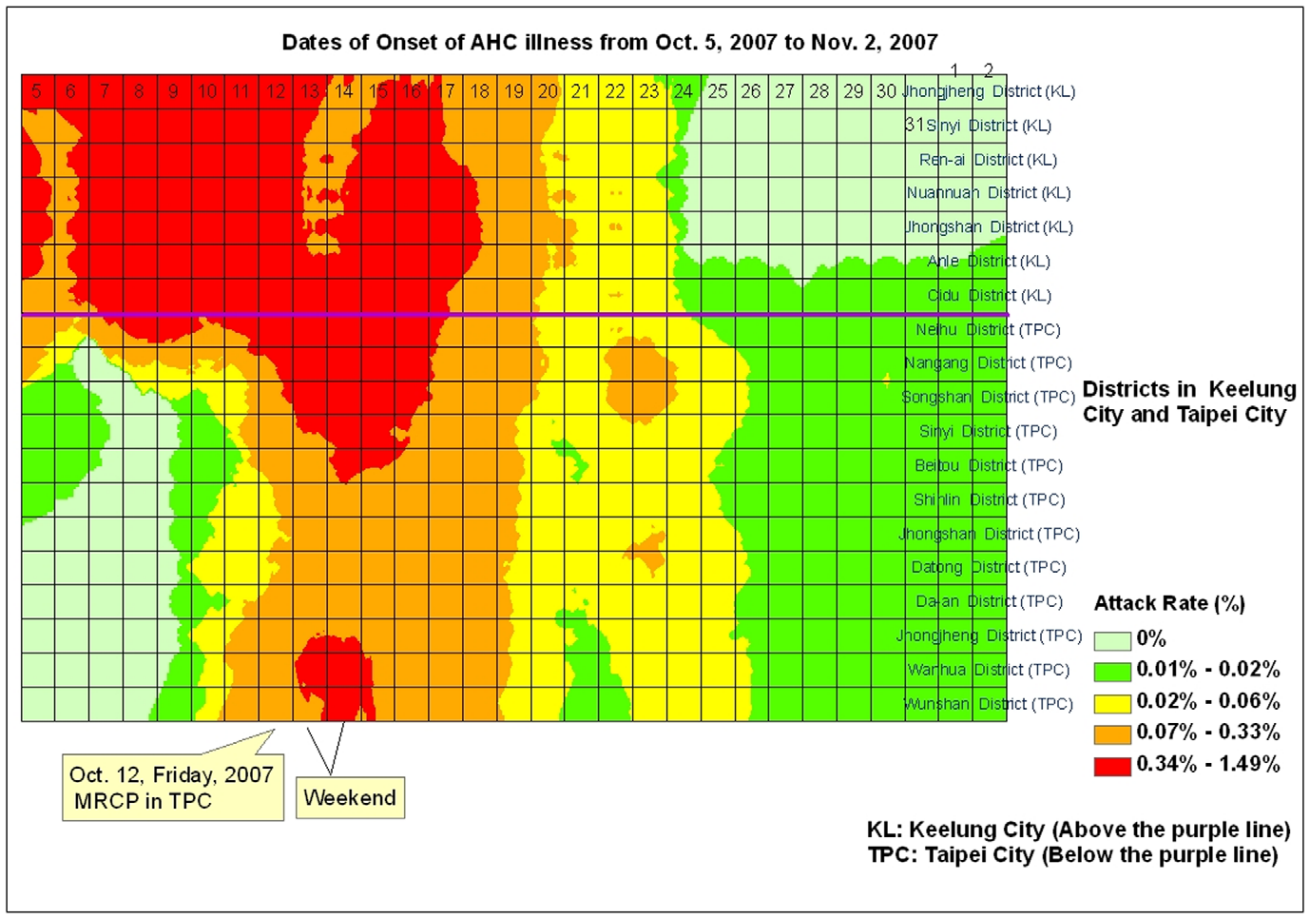
Spatio-temporal Diffusion Patterns of AHC Outbreaks from Keelung City to Taipei City, 2007. The X-axis reflects the temporal scale, while the Y-axis displays district names ordered by geographical correlation from North to South, East to West. The horizontal purple line marks the border between Keelung City and Taipei City.

The greatest number of AHC cases in Taipei City occurred on a Friday (October 12), signaling an upcoming weekend spike in infections. However, on that day, Taipei City government launched the multi-channel risk communication program, greatly reducing the incidence rate and, in effect, causing a sharp weekend drop in new cases. Because of such measures, the epidemic ebbed much earlier in Taipei than in Keelung (Figure 3).

#### 2. Epidemiological characteristics

**Figure 4:**
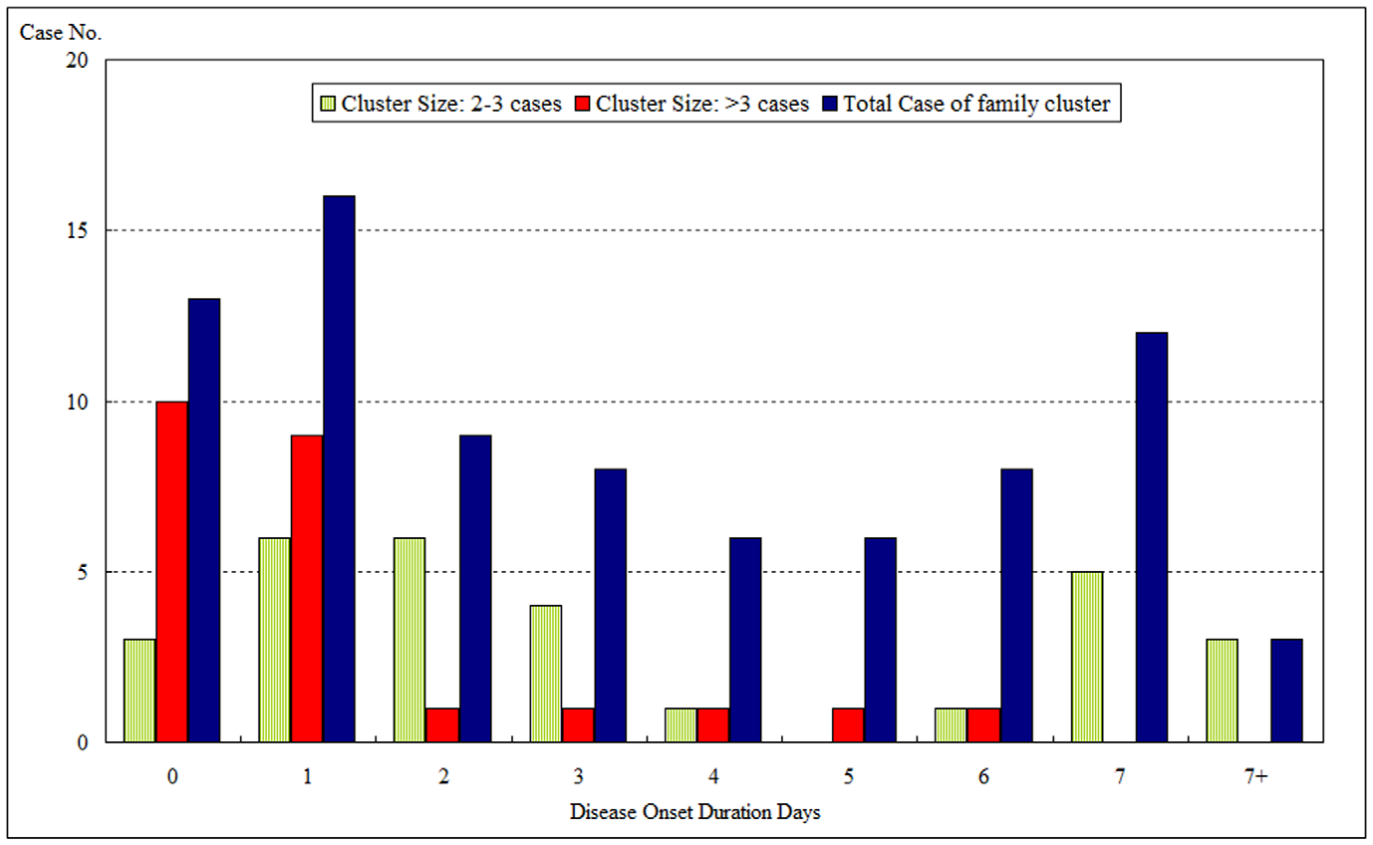
Distribution of AHC Illness Duration as Related to Family Cluster Size. Onset duration tended to be shorter when cluster size was more than 3 family members. (1.2±1.7 days, range 0 to 6 days, *vs.* smaller cluster size 3.9±4.0 days, range 0 to 16 days, p<0.01).

The outbreaks began in Keelung in September of 2007. Due to the high frequency of transportation from Keelung to Taipei, the epidemic gradually reached Taipei. Districts in Taipei City closest to Keelung City began to see an increase in their attack rates on October 8th (Figure 3). The wave of new infections moved steadily from the northeast districts to the southwest districts of Taipei. In both cities, the disease spread citywide within a short period (4 days), as shown in Figure 1, and the median time between exposure and onset of disease was as short as 1 day [mean ± standard deviation (SD): 2.6±3.2 days, range 0 to 16 days] [Figure 4]. The mean time from exposure to onset of AHC was significantly shorter in family clusters than in school cluster cases (mean ± SD: 2.6±3.2 *vs.* 4.39±4.82 days, p = 0.03). The mean and range of time between exposure and disease onset was also significantly shorter in larger family clusters (>3 AHC cases per family) than in smaller ones (< =  = 3 AHC cases) (1.2±1.7 days, range 0 to 6 days *vs.* 3.9±4.0 days, range 0 to 16 days, respectively) (p<0.01).

In terms of risk factors (Table 2), boys were at significantly higher risk for AHC-related pink eye than girls (OR = 2.14, p<0.001), and older children (over 10 years old) were generally at higher risk for pink eye both at home (OR: 2.56, p = 0.008) and at school (OR 3.51, p<0.001). In general, children at northeastern Taipei schools, located closest to Keelung, were at greater risk for pink eye (OR = 2.26, p-value = 0.003) both at home (OR = 3.93, p-value <0.001) and at school (OR = 2.46, p-value  = 0.001), than those at schools located in districts further away (Figure 1). In addition, school children were identified as the index cases in 75.5% of fifty-three family clusters (40/53), demonstrating their high risk for transmitting the disease to other family members.

**Table 2:** Demographical Information of Participating School Children Presenting/Not Presenting Pink Eye Illness (Within/Outside Family or School Clusters) during the AHC Outbreak in Taipei City, 2007.

	Red Eye Illness			Family Cluster			School Class Cluster		
	Present	Absent	OR	Yes	No	OR	Yes	No	OR
**Gender**									
**Male**	55	164	**2.17** *	32	187	1.45	53	166	**2.15** *
**Female**	28	181		22	186		27	182	
**Age (Years)**									
**<10**	15	128		10	133		12	131	
**≧10**	68	211	**2.75** *	45	234	**2.56** *	68	211	**3.51** *
**School Grades**									
**1–3**	5	131		4	132		2	134	
**4–6**	21	65	**8.46** *	13	88	**4.87** *	21	65	**21.64** *
**7–9**	57	181	**8.25** *	38	200	**6.27** *	58	181	**21.47** *
**School Areas**									
**Northeast**	63	215	**2.26** *	47	231	**3.93** *	62	216	**2.46** *
**Southwest**	21	162		9	174		19	163	
**Index Case in Family**									
**1st case**	61	11	**11.71** *	40	32	**4.13** *	59	13	**9.58** *
**Not 1st case**	18	38		13	43		18	38	

*p **<0.05.**

### B. Effectiveness of the Mass Risk Communication Program

#### 1. Home confinement of symptomatic school children

On Friday, October 12th (a day marked for its dramatic spike in new cases) the multi-channel mass risk communication program was launched in Taipei just prior to the weekend. On that day, three containment measures were urgently implemented: (1) the mayor addressed the press about the outbreak, (2) a letter written by the Taipei City Department of Health and signed by the mayor was given to students to take home to their parents, and (3) SMS messages were sent to all Taipei citizens with mobile phones. The collective message of all three channels emphasized the severity of the outbreak and outlined preventive measures, including home confinement. According to results obtained from the questionnaire (Table 3), home confinement of symptomatic students increased from a rate of 10% prior to the implementation of the risk communication program to 60% afterwards (p<0.05).

**Table 3:** Absentee Rates of Schoolchildren Pre and Post MRCP in Taipei City, 2007.

AHC Onset Date	Cases with School Absenteeism	Cases Attended Schools	Absenteeism Rate (%)
**Before MRCP** †	3	26	10.34%
**After MRCP**	8	5	61.54%*

†Mass risk communication program (MRCP) was launched on Oct. 12, 2007 in Taipei City.

*p = 0.00116.

#### 2. Disease containment

As mentioned above, Taipei launched its mass risk communication program on October 12th. This was also the day that the epidemic reached its highest peak. As demonstrated in Figure 2, the pre-weekend case surge was reduced by almost half by October 15th (following the mass risk communication campaign in Taipei).

In Keelung, where no such risk communication program was launched, the epidemic lasted for thirty-four days and had fourteen days of high attack rates within that period. In contrast, the epidemic lasted thirteen days in Taipei and had less than five days of high attack rates following the implementation of the risk communication program.

The diffusion map in Figure 3 further illustrates the limited scope and short duration of high AHC attack rates (labeled in red) in Taipei districts. By October 17, attack rates in all Taipei districts reduced to moderate levels (labeled in yellow) and then further reduced to low levels (labeled in green) on October 26 (Figure 3).

#### 3. Sources of information related to control measure compliance and satisfaction evaluation of the SMS program

According to Taipei City's parents' responses to the questionnaire, primary sources for AHC information included TV news broadcasting (54.45%), daily school health education programs during morning assemblies (34.92%), and the Taipei City Department of Health letter signed by the Mayor (29.28%) (Table 4). Approximately fourteen percent (14.32%) of parents surveyed identified the short message service (SMS) issued by the Taipei City Department of Health as their primary source of AHC information.

**Table 4:** Parents' Primary Sources for Control Measure Information from Taipei City Government.

Information Sources Knowledge Content (%)	Absent from School and stay Home while Pinkeye (N = 461)	Household Segregation for Pinkeye Illness (N = 461)	Household Environmental Disinfection (N = 461)
**TV Broadcasting**	54.45%	50.54%	47.94%
**Schools' Prompt Daily Morning Assembly Education Program**	34.92%	34.49%	35.79%
**Letter to Schoolchildren's Parents**	29.28%	27.98%	28.42%
**Newspaper**	24.95%	23.21%	22.34%
Mobile Phone SMS*	14.32%	10.20%	10.85%
**Parent's own Decision (from Regular Public Health Resources)**	7.81%	19.52%	22.56%
**Radio News**	5.64%	6.29%	5.42%
**Taipei City Mayor's Announcements**	3.90%	4.77%	4.56%

Based on a five-point scale, parents who received the SMS communication felt more satisfied with this method as a means of public health communication than those who did not receive SMS messages (3.89 *vs.* 3.01; p<0.05).

## Discussion

Prompt and effective prevention and control measures to combat EIDs are imperative to maintaining public health and safety. In this study, we found that the multi-channel risk communication program launched midway through the 2007 AHC epidemic in Taipei City increased the number of students confined to their homes, reduced total duration and affected areas of the epidemic, and decreased the number of dangerous, high attack days. By interrupting the prevailing transmission route between school and home, the program effectively inhibited the spread of this highly communicable disease in the community.

AHC has been identified as a highly contagious disease, capable of far-reaching, epidemic spread, since its first reported case in 1970 [2], [14] The Coxsackie virus A24 (CV-A24) variant in particular has been the causing agent of several difficult outbreaks. It has also been a significant challenge to sensitivity and timeliness efforts in disease surveillance systems [13], [15]. In addition, the virus's eye-related symptoms [16], [17], [18] are not easily differentiated from other infections (i.e. the human strain of Netherland's 2003 avian influenza, H7N7) [19], [20], [21], [22]. During the 2007 AHC epidemic in Taiwan, schools were found to be epicenters of transmission. By focusing disease control efforts in the school system, Taipei was more effective than neighboring Keelung (which relied on traditional control strategies) at interrupting the school-family-community cascade of transmission.

Because AHC was not initially listed as a reportable disease when this outbreak first occurred, accessible data was initially unavailable for cases before October 4, 2007. Several characteristics of the 2007 outbreak suggest a foreign source for Taiwan's AHC epidemic. The introduction of the outbreak in Keelung, a port city, and the genotype II status of the 2007 CV-24 virus (all CV-24 viruses isolated in Taiwanese outbreaks prior to 2007 had belonged to genotype III [13]) strongly suggest that the virus was imported in the summer of 2007. The spread worsened in Taiwan after schools returned to session in September. Although health officials in Keelung had advocated for hand washing, eye protection, and disinfection, new cases continued to rise after October 8. The CV-24 virus, spread primarily through person-to-person contact and contact with infected fomites, may also have spread through contact with respiratory droplets and fecal-oral routes, resulting in rapid and widespread transmission [13], [15]. Older children were more likely to be members of case clusters at home and at school. The reasons for this increased likelihood is unknown, though it may be related to higher rates of participation in team sports and/or less compliance with rules of hygiene. Research may be needed to fully understand this phenomenon. In essence, school children serve as index cases in around seventy-five percent of family clusters (Table 2) and are, indisputably, the largest transmitters of the virus. The findings of the study illustrate that schools serve as epicenters of CV-24 transmission and are effective intervention targets for containing the virus before its introduction to the general population.

The large weekend increases of CV-24 infection that occurred in Keelung may have resulted from a lack of preventive measures at home, e.g. ineffectiveness at keeping symptomatic children separate from other family members and/or improper disinfection of the home environment. Because the median time from exposure to onset is short (median 1 day in Figure 4), AHC is able to spread quickly from one index case to other family members, many of whom might take it back to schools and larger communities over the weekend. This creates a highly efficient family-school-community transmission cascade that is capable of spreading quickly throughout a city. Therefore, measures that effectively reduce and isolate infections at home and at school would contribute greatly to breaking the transmission cycle.

In the face of an unexpected outbreak of EID, risk communication and management are essential for keeping infection and mortality rates under control. The large disparity between mortality rates of the 1918 influenza pandemic in Philadelphia and St. Louis in the United States was due to distinct differences in the preparedness and timeliness of their respective local public health responses [23], [24]. This example serves to illustrate the importance of preventing and containing EIDs in a systematic and timely manner.

As the Capital of Taiwan, Taipei City has a much larger population size and density than Keelung City (Table 1). In addition, Taipei is a highly dynamic city with frequent economic, cultural, and social change. This inconstancy, coupled with its status as a large international travel hub, makes the city particularly vulnerable to EID. Much of Taipei's preparedness was the result of the city's experience as Taiwan's epicenter of the SARS outbreak in 2003. After the SARS outbreak, Taipei City Government initiated a new public health plan using an integrated infection control system against EID. This new system integrated early detection of outbreaks (particularly in hospitals and schools), epidemiological investigation, and epidemiologically based public health prevention and control policies. The renovated Division of Disease Control and Prevention (Taipei's CDC) also became the core operational unit for implementing crisis management procedures and facilitating policy. These systematic upgrades allowed for quick enactment of multi-channel risk communication measures during the 2007 outbreak of conjunctivitis.

The 2007 AHC outbreak provided the perfect opportunity for Taipei health authorities to test the effectiveness of the newly renovated system. Measures that were used to contain this epidemic may also, in turn, serve as a practice model for dealing with future influenza pandemics. There are many similarities between AHC and novel influenza: (1) there is currently no available vaccine for the circulating virus strain and anti-viral treatment is limited, (2) time from exposure to onset is very short, (3) the disease is highly contagious and easily contracted through contact with contaminated aerosols/droplets or fomites, and (4) schools serve as epicenters for the spread of the virus to households and, eventually, to the larger community.

It has been previously demonstrated that home confinement of symptomatic children can dramatically limit the spread of AHC and influenza in the community [25], [26], [27]. The timely launch of our multi-channel risk communication program to all Taipei citizens on Friday, October 12, allowed us to provide clear instructions to families on how to prevent the infection at home over the course of weekend, when infection rates would normally increase. These direct communication methods successfully convinced parents to keep their symptomatic children at home (from 10% to 60% in Table 3). With contagious students confined to their homes, school transmissions decreased dramatically and public health officials were able to contain the Taipei outbreak quickly. The effectiveness of these innovative methods in the heavily populated metropolitan area of Taipei City was evidenced by how quickly the epidemic, which had infected 5414 students, subsided after 2 weeks. This can be compared with the two months that were needed to control Taipei's previous CV-24 epidemic in 1987 [28].

The importance of appropriate risk communication in response to disasters has often been overlooked by public health officials in Taiwan. Traditionally, the mass media has helped disseminate epidemic information and increase awareness on how health risks may be reduced. However, the modern mass media has, at times, had a negative impact on public health efforts by encouraging public indifference or sensationalizing incomplete and inaccurate information. The media's reporting of the SARS outbreak contributed to unnecessary chaos in the early phases of the epidemic [29].

Rather than rely on the media alone to convey productive messages regarding the epidemic, Taipei's CDC was able to implement its Multi-Channel Mass Risk Communication Program to reach the public directly during critical points in the epidemic. We believe the SMS messaging component of the program was integral to the success of the 2007 AHC intervention and will continue to explore the use of this tool. We know from our questionnaire that fourteen percent of parents who confined their affected children at home during 2007's AHC outbreak reported that their decision to comply with this preventive measure was based on the SMS messages they received. Although the effects of the cell phone method cannot be fully isolated from the multi-channel risk communication system in this study, we believe that future interventions that utilize SMS exclusively will provide more insight on the effectiveness of this method. Taipei's CDC has since conducted a SMS campaign on Chinese Valentine's Day in 2009 to reach high-risk groups for Human Immunodeficiency Virus (HIV). The message asked the public to answer an AIDS-related trivia question and also provided information on free, anonymous HIV testing. After this exploratory campaign, Anonymous HIV screening rates went up 20% from the same time the year before. As SMS messaging is still an emerging approach to wide-scale information dissemination, we believe that further examination into the effectiveness of cell phone-based risk communication methods will need to be done at the local, national, and international level.

Taipei's ability to launch such a large-scale SMS campaign was a direct result of Taiwan's Communicable Disease Act (2006). This act allowed government officials to override the people's right to privacy when responding to epidemic disasters. In this case, the Taipei city government held a contract with Taiwan's six major mobile phone companies and committed all of them to allowing six free public service messages (per year) to be sent to their users if deemed necessary by the proper authorities. The SMS message in response to the AHC epidemic was sent to 2.2 million registered mobile phone users in Taipei City. Public satisfaction with the SMS campaign was high, especially amongst parents of school children. While this mass communication method has been advocated for use in many Asian countries, Taiwan's large-scale employment of SMS technology was, to the best of our knowledge, the first such attempt in the world. While it was, overall, an effective means of communication, the efficacy of this method was limited by several factors. First, the sudden influx of over two million messages put a large burden on the network system and resulted in a long delay (many received their message around midnight). Second, many Taipei mobile phones users are registered in other cities and did not receive the messages. Third, consumer weariness may have caused some people to ignore the long, unsolicited message before they read it. To reduce technical limitations, we suggest that SMS surge capacity be increased and tested at both non-epidemic and pre-pandemic stages. Other limitations to the SMS tool and alternative mass communication prevention methods can be fine-tuned with more experience, frequently updated guidelines, an efficient system for risk management, an integrated public health plan, and extra training for public health personnel [30], [31]. Another important challenge is reaching diverse populations, particularly those with low socio-economic status that may not be able to afford mobile phone service (and reside in less affluent, high-risk areas) [32]. This may be less of problem in Taiwan than in other countries.

This study had several limitations, including a lack of early data on pink-eye cases in Keelung. After the conclusion of the outbreak, there was also no further assessment on the effectiveness of control measures in Keelung. This study was non-randomized and missing epidemiological information for calculating secondary attack rates, age-specific asymptomatic ratios, and geographical diffusion of AHC cases outside Taipei and Keelung. Seroepidemiological studies in the future should be able to obtain more accurate secondary attack data with the use of comprehensive infection and disease exposure data.

In conclusion, the timely launch of the multi-channel risk communication program described in this study greatly reduced the duration and number of cases of Taiwan's 2007 AHC epidemic. These efforts effectively avoided a potentially large-scale epidemic of AHC in Taipei City. In encountering challenges such as outbreaks of influenza pandemic, or other EIDs with short incubation periods, public health officials need to prepare an integrated and timely administrative public health response. Urgent intervention and education must reach the community directly through multi-module channels, like SMS, for rapid communication.

Geographical variations in epidemiological characteristics, as described in our comparison analysis of the AHC epidemic in Keelung versus Taipei, are similar to variations in swine-origin H1N1 outbreaks in Mexico versus United States. Such similarities support this intervention's potential applicability to the prevention and control of other EIDs. Based on the findings of this study, we believe that the success of this risk communication method is dependent on: (1) the timeliness of the communication, (2) simplicity and consistency of the message, (3) appropriateness of the channels of dissemination, (4) transparency of the information, and (5) public faith in the communicator; in this instance the mayor of Taipei. While we found that the use of SMS significantly contributed to the effectiveness of the risk communication program, more SMS-based campaigns and research, like the HIV testing campaign mentioned in our manuscript, are needed to fully evaluate its effectiveness at communicating public health messages to the public.

## Supporting Information

File S1 Informed Consent and Questionnaire. Written informed consent was obtained before parents of the participated schoolchildren responded to the questionnaire.
(0.12 MB PDF)
