# Supplementary material for: Taipei's Use of a Multi-Channel Mass Risk Communication Program to Rapidly Reverse an Epidemic of Highly Communicable Disease
Source: PLoS One. 2009 Nov 23;4(11):e7962. doi: 10.1371/journal.pone.0007962 (PMC2776508; doi:10.1371/journal.pone.0007962)
Supplement: File S1 — Informed Consent and Questionnaire. Written informed consent was obtained before parents of the participated schoolchildren responded to the questionnaire. (0.12 MB PDF) [file pone.0007962.s001.pdf]

九十六年臺北區學生結膜炎(紅眼症)監測問卷

問卷填寫者（家長）:__________ 填寫日期：___月___日

親愛的家長您好：

有鑒於結膜炎(紅眼症)在全國各地引起大流行，且造成家長們的恐慌，因此臺北市政府衛生局特製此問卷以了解紅眼症相關資訊。此問卷將**作為防疫決策之參考**，不會將您隱私的資料洩漏出去，敬請放心填寫。臺北市政府衛生局感謝您的配合與協助，並敬祝您闔家平安，順心！

臺北市政府衛生局 敬啟

一、學生(個人)基本資料

1.姓名:__________ 班級：___年___班 性別:□1.男 □2.女

2.出生日期:民國___年___月___日 年齡:_____歲

3.出生地: □國內 _____縣/市______鄉鎮市區 □國外_______

4.現居地址:_____縣/市______鄉鎮市______區_____里/村______路街____巷____弄

5.是否已就醫 □是，(1)_______醫院；(2)_______診所；(3)_______校護(4)其他_______；
□否

6.發病前是否因其他眼疾去過眼科診所？□(0)無 □(1)有 □(9)不知

二、流行性結膜炎流行病學資料

1. 學生同住的家人中，是否有人得紅眼症，共______位患者，若有請填下表：

發病學生與家人（以發病次序填寫）	與學生之稱謂	年齡	發病首日（月/日）	發病結束日（月/日）	何處感染			
					同事	同學	家人	未知
學生本人								
家人1.								
家人2.								
家人3.								
家人4.								
家人5.								

2. **請問您貴子弟若有紅眼症**，在發病一週內是否接觸過**類似「紅眼症」症狀的個案（未發病者免答）**

□ (0)否

□ (1)是，關係是：□家人_____；□朋友；□同學；□鄰居、其他______；共_____人

□ (9)不清楚

(a)學生若有紅眼症，是否為**班上第一位**感染者？□(0)否 □(1)是 □(9)不清楚

(b)學生若有紅眼症，是否為**家中第一位**感染者？□(0)否 □(1)是 □(9)不清楚

(c)學生若有紅眼症，除了班上、家中以外在**其他社區活動地**(如圖書館、電影院、KTV、游泳池)，個案本身是否有見過紅眼症病例？ □(0)否 □(1)是 □(9)不清楚

3. 請問您貴子弟在紅眼症發病後，有無請假？

□ (0)無；

□(1)有（請假___天）；

□(9)不清楚

4. 請問您是從何處得知紅眼症須要在家休息，盡量不要上學的資訊（可複選)：□(1)手機簡訊 □(2)電視報導 □(3)台北市市長 10 月 12 日記者會 □(4)報紙 □(5)收音機廣播 □(6)學校之疫情宣導□(7) 台北市衛生局與教育局所發布之「給家長的一封信」□(8) 家長自行決定 □(9)身體不適因而無法上學 □(10)其他，請註明：________

5. 請問您貴子弟請假而未上學的決定是基於下列一項資訊(可複選）（**未發病者免答**)：□(1)手機簡訊 □(2)電視報導 □(3)台北市市長10 月 12 日記者會 □(4)報紙 □(5)收音機廣播 □(6)學校之疫情宣導 □(7) 台北市衛生局與教育局所發布之「給家長的一封信」□(8)家長自行決定 □(9)身體不適因而無法上學 □(10)其他，請註明：________

6. 請問您貴子弟發病後在家中休息時，是否有注意居家環境消毒（**未發病者免答**）？

□ (0)無；

□ (1)有；

□ (9)不清楚

7. 請問您貴子弟發病後在家中休息時，是否有注意與家人做基本的隔離（例如：不共用毛巾、洗臉盆…)（**未發病者免答**）？

□ (0)無；

□ (1)有；

□ (9)不清楚

8. 上述在家消毒知識資訊來源(可複選)：□(1)手機簡訊 □(2)電視報導 □(3)台北市市長10 月 12 日記者會 □(4)報紙 □(5)收音機廣播 □(6)學校之疫情宣導 □(7)台北市衛生局與教育局所發布之「給家長的一封信」 □(8)家長自行決定 □(9)平常學校的腸病毒教育□(10)其他，請註明：______________________

9. 上述在家隔離知識資訊來源(可複選)：□(1)手機簡訊 □(2)電視報導 □(3)台北市市長10 月 12 日記者會 □(4)報紙 □(5)收音機廣播 □(6)學校之疫情宣導 □(7)台北市衛生局與教育局所發布之「給家長的一封信」 □(8)家長自行決定 □(9)平常學校的腸病毒教育□(10)其他，請註明：______________________

10. 請問您貴子弟是否參與下列學校活動？(可複選)

□否；

□ (1)是：□(a)電腦課 □(b)音樂課 □(c)球類活動 □(d)游泳 □(e)其他運動，請註明：________ □(f)家政課□（g）實習工廠□（h）工藝課□（i）其他專科教室，請註明：________

11. 請問您貴子弟是否有參與**課外/課後活動**?(可複選)

☐ (0)否 ;

☐ 是:☐(1)安親班 ☐(2)補習班 ☐(3)校隊 ☐(4)社團 ☐(5)其他________

12. 請問您貴子弟班上是否曾有**相同症狀**的個案出現

☐ (0)否;

☐ (1)是:**共_____人**;

☐ (2)不清楚

13. 請問您貴子弟在發病前 2 週是否到過居住外的其他縣市見過紅眼症個案(**未發病者免答**)

☐ (0)否;

☐ (1)是:_____縣市,如有見過,第幾___天發病

14. 請問您貴子弟最近一週內是否搭乘過**大眾運輸工具**

☐ (0)否;

☐ 是:☐(1)公車 ☐(2)捷運 ☐(3)火車 ☐(4)高鐵 ☐(5)長途客運 ☐(6)計程車 ☐(7)飛機 ☐(8)其他____

15. 請問您貴子弟平常是否有正確洗手的習慣

☐ (0)否;

☐ 是:☐(1)會洗手,但不是每次 ☐(2)會定時洗手,但沒有正確洗手 ☐(3)都會按照「濕搓沖捧擦」的步驟正確洗手

16. 請問您貴子弟是否喜歡到處觸摸物品

☐ (0)否;

☐ 是:☐(1)偶爾 ☐(2)常常 ☐(3)總是

17. 請問您貴子弟是否到處觸摸物品後會洗手

☐ (0)否;

☐ 是:☐(1)偶爾 ☐(2)常常 ☐(3)總是

18. 請問您貴子弟是否在觸摸完物品後會去揉眼睛

☐ (0)否;

☐ 是:☐(1)偶爾 ☐(2)常常 ☐(3)總是

19. 請問您老師是否定期檢查學生攜帶手帕與衛生紙

☐ (0)否;

☐ (1)是;

☐ (9)不清楚

20. 請問您貴子弟過去是否曾有下列疾病?(可複選):☐(0)均無 ☐(1)結膜炎(非此次紅眼症) ☐(2)角膜炎 ☐(3)砂眼 ☐(4)白內障☐(5)弱視 ☐(6)乾眼症 ☐(7)視網膜受損

□(8)其他_______

21.請問您貴子弟此次發病症狀有(可複選)(**未發病者免答**)：□（1）結膜充血 □（2）眼睛刺痛 □（3）分泌物變多 □（4）怕光 □（5）灼熱 □（6）容易流眼淚 □（7）異物感 □（8）看不清楚 □（9）局部淋巴腫大 □（10）其他_______。

22.請問貴家長是否有收到此次防疫手機簡訊？

□（0）否；□（1）是

23.請問您是否滿意此次臺北市政府以手機傳遞防疫簡訊資料？

□（0）非常滿意；□（1）滿意；□（2）普通；□（3）不滿意；□（4）非常不滿意
